# Traumatic past in the present: COVID-19 and Holocaust memory in Israeli media, digital media, and social media

**DOI:** 10.1177/01634437211036997

**Published:** 2022-04

**Authors:** Liat Steir-Livny

**Affiliations:** Sapir Academic College, Israel

**Keywords:** COVID-19, digital culture, Holocaust, media, memory, social media

## Abstract

The COVID-19 pandemic began in 2019, spread to the rest of the world in 2020 and still holds nations in its grip in 2021. There is scant research on the way it has affected Holocaust awareness. Based on scholarly work on Holocaust awareness in Israel, the top-down memory of the Holocaust in the media and the vernacular Holocaust memory on social media, this article analyzes the ways the Holocaust became a frame of reference in Israel for the interpretation of the COVID-19 pandemic and the newest link in a long chain of using the Holocaust as a prism for other topics in Israeli society. The article centers on the evolution of the COVID-19 – Holocaust references and the role of media and social media in it. It shows that the initial panic created a wave of comparisons between the Holocaust and the pandemic in the media and social media. In the second half of the year, as the restrictions and two more lockdowns became part of life, references to the Holocaust changed – negative reactions to COVID-19 government regulations and law enforcement were compared on social media to Nazi acts. The Israeli media did not create these comparisons but reported them widely and contributed to their circulation.

The COVID-19 pandemic began in 2019, spread to the rest of the world in 2020 and still holds nations in its grip in 2021. The scant research on the way it has affected Holocaust awareness has mostly focused on Holocaust museums and memorials (Ebbrecht, 2020; Walden, 2020) and the emotional effects of the pandemic on survivors (Bagno, 2020; Wurgaft, 2020). Based on scholarly work on Holocaust awareness in Israel, the top-down memory of the Holocaust in the media and the vernacular Holocaust memory on social media, this article analyzes the ways the Holocaust became a frame of reference in Israel for the interpretation of the COVID-19 pandemic and the newest link in a long chain of using the Holocaust as a prism for other topics in Israeli society. This article centers on the ways in which references to the Holocaust changed as the pandemic waxed and waned in Israel and the role of media and social media in this evolution. It shows that the initial wave of panic in February 2020, followed by the first restrictions and lockdown, and the fact that Holocaust Martyrs’ and Heroes’ Remembrance Day was commemorated in April created multiple comparisons between the pandemic and the Holocaust. In the second half of the year, as the restrictions and two more lockdowns became part of life, references to Holocaust altered considerably. Israeli Jews realized that COVID-19 could not be equated with the sufferings of the Holocaust or the resilience of the survivors and veered toward different Holocaust associations that included comparing COVID-19 related government regulations to Nazi rule. Israeli media did not create these comparisons but reported them widely and contributed to disseminating the distortion.

## Holocaust awareness in Israel: media and social media

The Holocaust remains a central trauma in Israel’s national consciousness. The memory of the trauma has not faded over the years; on the contrary, Holocaust representations and the public discourse on the Holocaust have grown stronger since the 1980s for various reasons. For example, the right-wing Likud Party, which rose to power in 1977, made the Holocaust a permanent feature of high school curricula and matriculation exams since the 1980s. High school delegations first began to travel to former concentration camps in Poland in 1988. Today, myriad of Israeli students go on guided trips each year in 11th or 12th grade. These trips have become one of the most intensive ways of educating future generations about the Holocaust (Feldman, 2008). At the same time, in the 1980s, many Holocaust survivors retired and began to write their memoirs. This was also when second-generation survivors entered the cultural sphere and increased Holocaust awareness by expressing their parents’ trauma and their postmemory (Hirsch, 1996) in fiction and nonfiction, poetry, films, the theater, media, etc. The third generation started providing its own sense of the trauma starting in the late 1990s. Many Israeli Jews who are not related biologically to Holocaust survivors also engage viscerally with the trauma. Nevertheless, in the last 20 years a growing number of Israeli Jews have begun to criticize elements of canonical Holocaust commemorations, such as the intense acting-out of the trauma, and the instrumentalization of the Holocaust (Ofer, 2009; Porat, 2011; Steir-Livny, 2019). Some voice their critiques through previously taboo genres such as black humor, satire, and parody (Steir-Livny, 2017).

Holocaust awareness and discourse are not limited to the Holocaust as a historical event. Since the establishment of the State of Israel mentions of the Holocaust and Holocaust terminology have remained an integral part of public discourse on unrelated topics such as security, politics, and ethnicity (Steir-Livny, 2014). These associations have intensified as Holocaust awareness has deepened. For example, since the late 1970s there has been a growing tendency on both the Right and the Left to politicize the Holocaust to suit their opposite agendas (e.g. Bar-Tal, 2007; Zertal, 2002). Studies analyzing this extensive use of Holocaust memory in contemporary Israel have argued that the media, the educational and cultural arenas, and public discourse frame the Holocaust as a current, ongoing local trauma rather than as an event that ended decades ago in another place (Meyers et al., 2014).

Alongside the widespread visibility of the Holocaust in Israeli media, Holocaust institutes, and memorial sites make considerable use of social media for Holocaust commemorations. Analyses of representations of Holocaust memory in user-generated online platforms and user-generated content (UGC) employ the term ‘vernacular memory’ to describe this type of memory, which unlike official memory derives from the public space and is a reflection of ‘psychological, social, linguistic, and political processes that keep the past alive without necessarily intending to do so’ (Stangl, 2008: 246). Vernacular memory is non-hierarchical, popular, informal, unplanned, and sometimes subversive (Yadlin-Segal, 2017: 30–31). It serves as a ‘milieu for social action and as loci of oppositional collective memory’ (Stangl, 2018: 246). User-generated content has become an important part of vernacular memory which testifies to way ‘individuals negotiate, reconstruct, and share their versions of a collective memory in a given context’ in online web-based commemorations (Yadlin-Segal, 2017: 30–31). Vernacular memory thus constitutes another layer of representations, associations, and meanings to collective memory.

Recent works on aspects of vernacular Holocaust memory in social media have analyzed how individuals implement representations of Holocaust museums and memorial sites (Bareither, 2021), the way local social media projects affect Holocaust memory in specific countries (de Bruyn, n.d.), the ethics of Holocaust representations on social media (Walden, n.d.a), and the framing of Holocaust popular knowledge on Wikipedia by web users (Wolniewicz-Slomka, n.d.). Research on Holocaust associations in Israeli social media has highlighted the way they are intertwined with the daily lives of Israeli Jews (Friesem, 2018; Steir-Livny, 2020). Associating the Holocaust with the COVID-19 pandemic in media and social media is the most recent link in a long chain of Holocaust associations with other tense, emotionally laden topics.

### Comparing the pandemic to the Holocaust

COVID-19 has prompted a global rise in Antisemitism. Multiple articles published in the Israeli media (print and digital) and a widely disseminated 2020 report by the Kantor Center for the Study of Antisemitism show that the pandemic has been a catalyst for numerous anti-Semitic accusations intertwined with anti-Israeli sentiment. These include claims that the Jews are to blame for the outbreak of the Corona virus by poisoning wells or by deliberately coughing, that the Jews caused the plague, that the pandemic is part of the Jews’ plan to take over the world by spreading the virus to undermine the global economy and society, and then selling a vaccine or medicine worldwide at huge profits. The litany continues with innuendoes such as that the Zionists are to blame for the spread of Corona, the Zionists are deliberately infecting the Palestinians with Corona, and that the ‘Zionist pandemic’ (i.e. the founding of the State of Israel) broke out in 1948 (Kantor Center, 2020). The Kantor Report showed that more than 40% of young Jews in Europe have considered emigrating in response to the rise in Antisemitism on the continent, and that there were 18% more serious anti-Semitic incidents around the world compared to last year (Kantor Center, 2020).

The combination of the rise of age-old antisemitic accusations against Jews, the restrictions on flights and traveling abroad, as well as the waves of lockdowns have all created a siege mentality that has heightened the shadows of the past. In a society drenched in Holocaust awareness, in which Israeli Jews are surrounded by Holocaust associations and where many interpret their private lives through Holocaust associations (Friesem, 2018; Yadlin-Segal, 2017) whether jocularly or in terms of its lasting trauma, it is no wonder that the stay-at-home orders, the need for social distancing and the growing number of dead all triggered the media and the social media to interpret the new world of the pandemic through the lens of the Holocaust in the first months of the outbreak.

Since March 2020 Israel had gone through three lockdowns where stores, the education system, cultural institutions, restaurants, cafes etc., were closed and Israelis were told to stay at home, only leave the house for essential reasons, not to congregate in groups of more than 10 people, and not to travel between cities. ‘Corona is a ghetto’ wrote journalist Shaked (2020). ‘There is nowhere to run. You cannot go abroad, and to escape Corona I’d have to find a smuggler who would take me in the dead of night to some European shore, just to discover that it had been conquered as well by Corona forces’. Laughter was recommended as an antidote, as in the past. The headline of an article which reviewed a comedy act produced before the lockdown emblematically announced: ‘Like the entertainers in the Warsaw ghetto, I attended the last stand-up show in Israel’ (Muscle, 2020).

The first person in Israel to die from Corona (March 20, 2020) was a Holocaust survivor. Poignant articles about him and interviews with his family members made the Holocaust-COVID connection (Hason, 2020). In general, the media reported on the way the lockdown and quarantines caused the trauma of the past to resurface among Holocaust survivors (Alon, 2020) or increased their sense of loneliness (Kan News, 2020). Holocaust survivors’ perceptions of the pandemic, their comparisons between the period and simultaneously their explanations why the present is different than the past were widely reported (Rozenshtein, 2020). As unemployment grew exponentially (Gams, 2021; Globes Team, 2021) along with concomitant cases of depression (Joyful, 2020), domestic violence (No Author, 2020a; Avni, 2020; Gil-Ad, 2020), and a rise in the numbers of sick and dying, media articles intended to make it clear to Israeli Jews that in comparison to the Holocaust, they had nothing to complain about. In fact, however, the articles themselves constituted another comparison: this was the comfort a Holocaust memory-stricken society had to offer.

For example, remarks by survivor Avraham Roth were published in the popular *Haaretz* newspaper:

And here we are again locked down at home, in small and sometimes isolated rooms. We, who have been hiding for years, find ourselves as then, completely dependent on the good will of caregivers, relatives or neighbors, who provide medicine and food, and do not know what will happen next [. . .] In the Netherlands, more locals betrayed Jews and handed them over to the Gestapo than locals who hid and saved Jews. Today, by contrast, we have nothing to fear. We just need to make sure that we will be tested (for Corona) and will get the proper medical care (Ruth, 2020).

In one TV sequence on four survivors, one focused on his loneliness while the other three talked about their lives during the Holocaust to highlight that the current situation was incomparably better. Buchenwald survivor Naftali First showed a picture of himself at the liberation of the camp to make it clear that despite the comparisons between then and now, his suffering then was much worse than what Israelis were being forced to cope with (Solomon, 2020). The Facebook page of *Yediot Aharonot* (2020), one of most widely circulated newspapers in Israel, published a post by Holocaust survivor Arie Nativ, age 85:

I survived the Holocaust and saw much worse things than Corona. After the ghetto was liquidated, we lived for a year and a half in a small basement underground without going out once. We did not see the sunlight, the sky, the stars, we did not breathe fresh air [. . .] so to see Israelis who go to the beach and flaunt the lockdown orders infuriates me [. . .] I sat in a cramped basement with more than 30 people, today people are in their own homes, with their loved ones, TV, food, and they are still complaining.

This post received 10,000 likes and was shared over 1500 times. Numerous responses indicated that the comparison appealed to Israeli Jews, who agreed with him, wrote that the lockdown had made them think more than ever about the Holocaust and the ways people survived, and criticized the current generation for being spoiled. In other cases, the contentious term ‘Corona Deniers’ was coined to castigate those claiming that the pandemic was simply a case of the flu. This play on words with ‘Holocaust deniers’ was considered by others to be in semantic bad taste (Arenstein, 2020).

Holocaust Martyrs’ and Heroes Remembrance Day (April 20, 2020) prompted even more such comparisons. People posted their reflections on the comparison between Corona, the lockdown, social distancing and the Holocaust. For example, *Beit Hamidrash Kolot* (Kolot Beit Midrash, 2020) published a post on Facebook saying that although Jews need to stay protected in their homes, now they can manifest their Judaism openly so that the present cannot be compared to the past when Jews had to hide in their homes but also hide their Judaism. A number of posts and blogs discussed how Holocaust survivor grandparents can provide ‘a lesson in proportions’ (see Shenzhen, 2020). A social worker discussed the growing incidence of Holocaust anxieties linked to Corona especially on Martyrs’ and Heroes’ Remembrance Day (Breslav, 2020). A clinical psychoanalyst prepared a Zoom lecture on the diaries of the Dutch writer Eti Hilsom, who was murdered in the Holocaust, from a Corona perspective. This included such comments as ‘To look through the window that Hilsom found into the sky inside of her in terms of the reality in the Corona era’, to ‘focus on the healing power of creation in the face of the most difficult existential challenges’ (No author, 2020a).

Eventually the comparison between the pandemic and the Holocaust became so widespread that in the days preceding Martyrs’ and Heroes Remembrance Day, Channel 7 screened commentary by third generation Efrat Briner, a poet and one of the activists in the alternative commemoration *Remembering in the Living Room* [in Hebrew: Zikaron BaSalon]. She asked the public to stop comparing the pandemic to the Holocaust:

As a Third Generation who grew up on stories of hunger, death and terror I am appalled to hear people comparing life during Corona to what my grandparents went through a few decades ago. When our refrigerators are full and social distancing is for the purpose of protecting us, when we have a country that works tirelessly to give its citizens the best care [. . .] how do we have any right to compare? [. . .] Staying at home with the children is not separation through selection for life and death. The desire to see the world is not death marches. Maintaining a distance of two meters in the supermarket is not starving to death. The masks and gloves we are forced to wear are not the shoes and glasses left *there* in piles. The comprehensive disinfection we do every few hours is not the showers in the gas chambers. The lockdown imposed on us is not a ghetto. Our lives is completely different [. . .]

She pleaded with the public to stop using these comparisons which dishonor the memory of the Holocaust, the murdered and those who survived. ‘Do not compare the Corona pandemic to the Holocaust. It will be buried in the pages of history as just another event that affected the world’ (Briner, 2020). Her appeal, however, was disregarded.

Holocaust Martyrs’ and Heroes’ Remembrance Day in Israel takes place on the Hebrew date of the 27th of Nissan (in April or the beginning of May). A day before Holocaust Martyrs’ and Heroes’ Day, Ronen Flut, the Head of the Nof Hagalil municipality wrote:

Holocaust Martyrs ‘and Heroes’ Remembrance Day in the shadow of the Corona plague prompts me to contemplate what would have happened if the world had behaved *then* as *now*. What would have happened in the world, for the most part, if it had not been silent and indifferent to the spread of the cruelest virus history has ever known - the Nazi virus. A virus that spread in appallingly huge proportions, exterminated human beings and was not the result of eating bat soup or an accident in a lab in China. The Nazi virus was a calculated act, planned by humans who simply decided to eliminate the Jews.

He added that *then* the world did nothing to help the Jews, but now the world’s reaction does not matter anymore ‘because unlike in the Holocaust, today we have a state and we no longer need the lifeline of the nations of the world. Sometimes they are the ones who need us’. He concluded, ‘the Corona era, on the eve of the Holocaust and Independence Day ceremonies, actually enhances the strength and power of the State of Israel and the Israel Defense Forces’ (Flut, 2020). The pandemic, the Holocaust and the Zionist lesson on both became one.

One of the key ceremonies that day takes place at the Yad Vashem Holocaust museum. The ceremony is broadcast on TV and it is customary for the prime minister, the president and other officials to give speeches. On Holocaust Remembrance Day 2020 Prime Minister Netanyahu stated that ‘We mourn the Holocaust survivors who were recently taken from us by the Corona virus’. He said that the Holocaust is unique and yet highlighted the similarities between the past and the present and the differences derived from them:

We are dealing with a dangerous pandemic. In the ghettos and camps epidemics raged. The concentration of tens of thousands of Jews in a limited area caused countless victims [. . .] Today we have a national home, a strong state [. . .] At the same time Israel must always be responsible for its own destiny, to defend ourselves [. . .] Unlike in the Holocaust, in the Corona case we recognized the danger in time [. . .] (Alon and Eichner, 2020).

He then coupled the threat of Corona to his longstanding reference to the Iranian threat. The association between the Holocaust and the politics of destruction of the Jewish state has been politicized since the early days of the Israeli state. It intensified in the late 1970s under right-wing Prime Minister Menahem Begin and exists in many variants in right wing and left-wing debates (Bar-Tal, 2007; Steir-Livny, 2016). It is also one of Prime Minister Netanyahu’s best known battle cries. He is well known for his use of the Holocaust when he discusses the Israeli – Palestinian conflict, the Israeli – Arab conflict or the Israeli – Muslim conflict. In his years in office, he often stressed the parallels between Arabs and Nazis, Palestinians and Nazis, and the similarities between Iran’s nuclear threats and Hitler’s goal of exterminating the Jewish people. Netanyahu has repeatedly promoted the impression that Iran would be responsible for a second Holocaust. Left-wing Israelis often criticize these analogies. Netanyahu stated:

The lessons of the Holocaust require us to be constantly vigilant. The threats of annihilation of radical Islam led by Iran have not subsided in the Corona storm. They are still here, and we are more determined than ever to address them. The IDF and the security forces are now assisting civilians in the Corona crisis but make no mistake: we are more than ever committed to facing any danger - near our borders, and far from our borders.

In this cocktail of the Holocaust, the pandemic and the Iranian front, this speech was supposed to guarantee that unlike in the past, today the Jews would be saved because he was at the helm. ‘Comparing Corona to the Holocaust would not have occurred to any Israeli except Netanyahu’ proclaimed the well-known *Haaretz* journalist Alpher (2020) and added: ‘Seriously, compare Corona to the Holocaust? No, no Israeli citizen would do that. For the simple reason that it’s just in his head’.

But Alpher was wrong. From February 2020 onward, Israeli media and social media made it clear that the comparison went far beyond Netanyahu’s personal politicizations. ‘There is no resemblance between the Corona pandemic and the deliberate extermination of the Jewish people by the Germans and their collaborators’ wrote physician and right-wing journalist Arie Eldad in response to Netanyahu’s speech. ‘Mentioning Corona and the annihilation of the Jews in one breath is almost a kind of Holocaust denial. God forbid, I do not blame Netanyahu for that. It was just a foolish attempt to link current events with the past’ (Eldad, 2020). However, in the remainder of his article he did just that. He wrote about the way Israel is fighting the plague, people who do not follow government orders because they have not adjusted their mindset to the new regulations, and that ‘the most annoying of all are the ideological refusers: the plague deniers’. He listed their explanations and wrote ‘and there are also all sorts of Holocaust deniers’ and then went to list their ‘explanations’ (Eldad, 2020).

### Corona regulations and law enforcement as Nazism

In his book *Holocaust Icons: Symbolizing the Shoah in History and Memory*, Baruch Stier (2015) analyzes several prominent symbols of the Holocaust such as railway carts, Anne Frank, the sign ‘Arbeit Macht Frei’, and the number six million. He argues that Holocaust icons are ‘certain symbols that have come to represent the Holocaust in encapsulated form [. . .] it is through the use of iconic symbols that the public meanings and perceptions of the Holocaust are created [. . .]’ (Baruch Stier, 2015: 2–3). Six months into the pandemic, Israeli Jews began to realize that COVID-19 was a dangerous virus but not a Holocaust. Restrictions, lockdowns and the siege mentality became routine. The comparison between the Holocaust and Corona toned down as the months passed. It did not completely disappear and, for example on International Holocaust Memorial Day, news articles about Holocaust survivors who compared their situation during the Holocaust and the pandemic resurfaced (Aidan, 2021; Yadid, 2021) but far more rarely. At this juncture, Israeli Jews began to exploit Holocaust icons to criticize law enforcement and government restrictions. Israeli Jews who protested governmental regulations used Holocaust-related associations, both visually and verbally.

For example, on December 21, 2020 the Corona task force decided to take people directly from the airport to Corona ‘hotels’ for immediate quarantine by bus. The decision was made so fast that most travelers found out about it when already in the air. A young woman who was interviewed on TV told the anchorman: ‘we were highjacked to Corona hotels. The elderly felt like they were in Auschwitz’ (No author, 2021a).

When many self-employed workers protested that the restrictions were taking away their livelihood, a spokesperson proclaimed in an interview on TV that ‘we will not go like lambs to the slaughter’ (Reshef, 2020). This famous battle cry was uttered by Aba Kovner in the Vilnius ghetto in January 1942. Kovner wanted to encourage the Jews to stand up against the Nazis and not surrender meekly. In the first decades after the Holocaust in Israel this slogan was turned against the survivors to suggest that in a society that embraced the myth of the brave and heroic ‘New Jew’, Diaspora Jews had not resisted the Nazis and went to their death without a whimper (Bar-On, 1997: 4). This judgmental mistaken notion has faded over the years, but the phrase in both senses is still considered to be one of the verbal Holocaust icons in Israel.

Ultra-Orthodox Jews in Israel have used Holocaust associations in many demonstrations and struggles against the secular state and its authorities. In numerous clashes between the Ultra-Orthodox and the Israeli police, shouts of ‘Nazis’ are a typical feature. The constant tension between secular Israel and Ultra-Orthodox society increased exponentially during the pandemic since many Ultra-Orthodox factions refused to comply with government restrictions. They refused to wear a mask and continued to attend crowded events at synagogues or weddings and funerals that attracted 1000s of people. During the three lockdowns when most of the educational system was shut down and children stayed at home for weeks, large sectors of the Ultra-Orthodox educational system remained open. When police tried to enter their neighborhoods or cities to verify the implementation of regulations they were met with violent demonstrations and constant screams of ‘Nazis’. In demonstrations against the restrictions on opening the educational system that took place in Jerusalem and the city of Beit Shemesh on Holocaust Remembrance Day 2020, the protestors even wore yellow badges (Forscher, 2020). Novick (2021), an observant TV anchor tweeted that the Ultra-Orthodox need to do deep soul-searching for their ‘Nazi’ taunts.

The refusal of Ultra-Orthodox sectors to apply the COVID-19 restrictions extended far beyond their own neighborhoods. In August 2020, the government of Israel asked the government of the Ukraine to close its borders to prevent 1000s of Hasidim from gathering at the annual celebrations on the Jewish New Year (September) at the grave of Rabbi Nachman in Uman. Many Ultra-Orthodox Jews who were stopped on the border between Poland and the Ukraine wore a yellow badge and were interviewed wearing it on Israeli TV.

The disregard of Corona regulations took a massive toll on Ultra-Orthodox society. In early 2021, the Ultra-Orthodox sector had the highest morbidity and mortality rate in Israel (Adamker, 2020; Linder, 2020). Yehuda Meshi Zahav, the head of the Ultra-Orthodox burial society ZAKA and a well-known public figure in the general media lost his mother to the Corona virus. In a conversation with the Ultra-Orthodox newspaper *Shabbat Square*, he begged the Ultra-Orthodox to adhere to health guidelines and stated that Corona deniers are worse than Holocaust deniers because ‘Holocaust deniers deny the past, and here it is a denial of the present’. The respondents in the talkback rejected this comparison (Rotman, 2021). A week later, Meshi Zahav’s father also passed away from COVID-19 (Ma’ariv Online, 2021).

Holocaust associations were also used in reverse fashion to criticize the behavior of the general public toward the Ultra-Orthodox. As the resentment toward Ultra-Orthodox conduct grew in the other sectors of the population, some claimed that this verbal mudslinging was a form of auto-Anti-Semitism. For example, Buzaglo (2020), a social activist, uploaded the famous picture of the girl in the red dress from Steven Spielberg’s 1993 film *Schindler’s List* to Facebook and wrote about the resemblance between attacks on the Ultra-Orthodox and the way the Nazis and their collaborators treated the Jews. He commented that when walking around in Bnei Brak (a major Ultra-Orthodox city) during the lockdown he saw a sad little girl ‘as sad as children shouldn’t be’ on a balcony and then he ‘remembered the red dress from Schindler’s List [. . .] and the nasty posts against the Ultra-Orthodox and the gloating like the Poles who smirked when they saw [the Jews] walking like lambs to the slaughter, and sorrow fell upon me’.

In January 2021 the government closed airports to all travel. Israelis who were abroad were stuck there and the government toyed with an idea of bringing them to Israel under the obligation to wear ankle bracelets to be sure they would not violate quarantine rules. One Israeli woman who was marooned in Dubai was interviewed on the Channel 12 evening news and explained she had flown there to celebrate her 64th birthday ‘which is on International Holocaust Remembrance Day’ and raged ‘I am a second generation to Holocaust survivors! You won’t put anything on me! I am not a criminal. My family members have numbers tattooed on their arm, who are you to put an ankle bracelet on me?’ (No Author, 2021a).

The first shipments of vaccines landed in Israel in December 2020, and the Corona cabinet began to discuss regulating obligatory vaccination and the possibility of issuing a ‘green passport’ to those who had received both doses. Confirming research which shows that social media platforms enable private citizens and opposition groups to take active roles in grassroots campaigns (Knobel and Lankshear, 2007: 1–24; Shifman, 2013: 119–50; Stache, 2015: 162–64) and that communicative spaces can create political and social awareness and encourage activism (Bennett and Toft, 2009: 246–60; Murthy, 2013: 92–114), anti-vaccine demonstrators uploaded various Holocaust associations to social media protest these decisions. A photo of the huge vaccine tent in Rabin Square in Tel Aviv was uploaded to social media with the caption ‘a Shot brother, a shot’ which is a play on words on the famous song ‘Our Town is Burning’ written by Yiddish poet Avraham Gebirtig to memorialize a pogrom in Poland in 1936. This melody was sung in the ghettos and concentration camps and has been performed at Holocaust commemoration ceremonies in Israel for decades (‘Our Town is Burning’, n.d.). Another picture uploaded to social media showed the dancing and celebrations in Tel Aviv Sourasky Medical Center (Ichilov) when the first doses of the vaccine were injected. The picture s began as a split screen also depicting Jews in the Holocaust who were forced to play musical instruments. The caption read: ‘they dance like sheep’. Posters in demonstrations, which were uploaded and shared on social media read ‘The last person to insist on compulsory vaccines was Josef Mengele’, ‘the green passport equals the yellow badge’, ‘The vaccine liberates’ and ‘Selection? No More’ (Chapnik, 2021). A picture shared on social media showed three masked figures with swastikas on their arms, with the figure in front raising her hand in a Nazi salute. The caption said ‘the Ministry of Health for a better life’. Journalist Nadav Eyal posted the responses he received on Facebook and Twitter when he reported in January 2021 that people from age 40 could get vaccinated (the vaccine operation initially began with the elderly). One of the responses was: ‘do you know you are complicit in Goebbels’ propaganda?’ On Facebook, vaccine opponents superimposed the heads of Minister of Health Yuli Edelstein and Netanyahu on pictures of Nazis in uniform with captions reading ‘If you won’t get the vaccine you will perish’. The Minister of Health’s last name (Edelstein) was altered to ‘Mengalestein’ as a distorted play on words on the most notorious Nazi Doctor Josef Mengele (Ungar, 2021). In another picture on Facebook the upper part of a picture uploaded to Facebook showed Israelis in Tel Aviv standing in line for Corona tests coupled with a picture of Jews in line in a concentration camp.

In February 2021, the Israeli government decided to issue a ‘green passport’ to people who had received a full vaccination. This granted entry to malls, cultural events, pools, gyms, etc., whereas unvaccinated individuals were barred. In response, a vaccine opponent posted a picture of Anne Frank on Facebook with quotations from her diary in which she describes the new restrictions against Jews in The Netherlands in June 1942, banning them from entering theaters, pools etc. (Leibowitz, 2021). ‘The comparisons between the vaccine campaign and the Holocaust are simply a type of Holocaust denial and Facebook, of course, is not dealing with it’ tweeted journalist Nadav Eyal and added a post of a woman who, like others, to protest the ‘yellow passport’, uploaded a picture of an arm marked with a product barcode and a caption that read: ‘there once was another country that marked a sector of its inhabitants – it was Nazi Germany’ (Eyal, 2021). The government’s special task force to root out fake news about Corona also reported on deniers’ use of Holocaust symbolism (Efrati, 2021).

Overall, the comparisons between the hardships of the Holocaust and the lesser hardships of the pandemic in the first half of the year switched in the second half of the year to comparisons associating government regulations and Nazism. Both of these uses point to the trauma of the Holocaust as a dominant reference in the identity of Israeli Jews across the spectrum. Politicians, public figures, ‘regular citizens’, people who obey the rules and people who denounce them, the secular and the religious, men and women of various ages, use the Holocaust as a frame of reference to deal with COVID-19 from various perspectives.

The combination of Holocaust memory and the pandemic in Israeli media, digital media and social media contributes to a better understanding of the depth of Holocaust awareness in Israel. It confirms research suggesting that Israel is a post-traumatic society which continues to act out the trauma of the past. The analysis of the top-down references in the media and the bottom-up vernacular memory on social media points to their prominent role in disseminating Holocaust associations. This is the newest link in a long chain of using Holocaust associations in Israel from 1948 to the present to discuss political, ethnic, social, and other issues. After more than 70 years, the process of working through the trauma is still far away.
